# Use of Focus Groups to Inform a New Community-Based Youth Diabetes Prevention Program

**DOI:** 10.3390/ijerph19159655

**Published:** 2022-08-05

**Authors:** Ngina McAlpin, Cordelia R. Elaiho, Farrah Khan, Cristina Cruceta, Crispin Goytia, Nita Vangeepuram

**Affiliations:** 1 Teen HEED Intern, Icahn School of Medicine at Mount Sinai, New York, NY 10029, USA; 2 Department of Pediatrics, Icahn School of Medicine at Mount Sinai, New York, NY 10029, USA; 3 Department of Neurology, University of Washington, Seattle, WA 98195, USA; 4 Teen HEED Community Action Board, Icahn School of Medicine at Mount Sinai, New York, NY 10029, USA; 5 The Institute for Health Equity Research, Icahn School of Medicine at Mount Sinai, New York, NY 10029, USA; 6 The Department of Population Health Science and Policy, Icahn School of Medicine at Mount Sinai, New York, NY 10029, USA

**Keywords:** youth, focus groups, community-based, diabetes prevention, peer education, mobile health technology

## Abstract

There have been few youth-led diabetes prevention programs. Our objective was to conduct focus groups to explore peer influences on adolescent lifestyle behaviors and strategies for implementing a youth peer education model for diabetes prevention. We conducted six focus groups with 52 youth (ages 13–22; 62% male, 38% female; 64% Hispanic, 36% non-Hispanic Black) from East Harlem, NYC. We used a Thematic Analysis approach to identify major themes, compared findings, and resolved differences through discussion and consensus. Three dominant themes arose: (1) Adolescents generally encounter more unhealthy peer influences on diet and more healthy peer influences on physical activity; (2) Adolescents endorse youth-led diabetes prevention strategies and describe ideal qualities for peer leaders and methods to support and evaluate leaders; (3) Adolescents prefer text messaging to monitor behaviors, track goals, and receive personalized guidance. Using study findings, our Community Action Board developed a peer-led diabetes prevention program for prediabetic adolescents.

## 1. Introduction

The prevalence in youth of type 2 diabetes, once thought to be exclusively a disease of adults, has increased by over 35% since 2001 [1]. There are also racial and ethnic disparities in diabetes rates with a higher prevalence of youth-onset type 2 diabetes among minority racial ethnic groups and an annual increase in diabetes incidence in all racial ethnic groups except non-Hispanic whites [2,3,4,5]. The potential public health and financial impacts of youth-onset diabetes are substantial since youth with type 2 diabetes tend to have higher rates and earlier onset of complications than adults, more aggressive disease, and less favorable response to treatment [6,7,8,9,10].

Prediabetes may quickly progress to diabetes, but the condition may be reversed if teens are able to maintain or lose weight [11]. Lifestyle interventions decrease risk of progression, [12,13] but there are few prevention programs for youth, especially for at-risk minority youth [14,15]. One strategy that has been found to be effective in at-risk adults is peer education [16,17,18]. Similarly, youth are more likely to modify behaviors if information is provided by peers, as exemplified in prior youth behavioral health interventions [19,20,21,22]. In addition, there is ample evidence that friends have a strong influence on diet and physical activity behaviors among youth [23,24,25,26,27,28,29,30,31,32]. However, there have been few youth peer-led diabetes prevention programs to date. In addition, researchers have not invited youth to help inform and develop programs designed to impact them.

Sustained engagement is another challenge with youth health interventions [33]. One promising strategy to keep youth engaged is incorporation of mobile health technologies such as social media, apps, and text messaging. The vast majority of youth (including minority youth) own and regularly use smartphones [34,35]. In addition, teens from lower-income households are more likely to use their phone to access health information than those from higher-income households [36,37]. Mobile health technology is therefore a promising strategy to engage vulnerable youth and address disparities. However, development of tools that are likely to be effective requires a deeper understanding of the types of platforms and content youth desire.

The objective of this study was to conduct focus groups with youth to explore peer influences on adolescent lifestyle behaviors that may impact diabetes risk, strategies for implementing a youth peer education model for diabetes prevention, and how best to incorporate mobile technologies as part of a youth diabetes prevention program.

## 2. Materials and Methods

### 2.1. Participants/Recruitment

The study was conducted in East Harlem, a predominantly low-income, mixed ethnicity neighborhood in New York City. East Harlem was home to an estimated 111,452 people in 2019 [38], with one-third of the population aged under 24 years [39]. Program directors at six community-based after school programs provided basic information about the study and identified interested adolescents and young adults (ages 13–22 years). Participants were recruited as per an Icahn School of Medicine at Mount Sinai Institutional Review Board approved protocol including written parent/guardian informed consent and participant assent for those <18 years and participant consent for those ≥18 years. We recruited 52 teens and held six focus groups at the collaborating community sites, with groups including a mix of younger and older, male and female, and Black and Latinx adolescents, representing the diversity of East Harlem. The average duration of the focus groups was 72 min. Ongoing data analysis indicated that theoretical saturation was achieved after completing six focus groups, so no further groups were conducted.

### 2.2. Data Collection

We employed a Community-Based Participatory Research (CBPR) approach to conduct this study [40]. Our community action board (CAB) is comprised of academic and community stakeholders including youth. We worked with the CAB to draft a focus group moderator’s guide to capture: (1) how social and environmental factors, knowledge, attitudes, beliefs, and behavioral influences affect teens’ lifestyle behaviors associated with diabetes risk; (2) ideas about youth diabetes prevention using peer education; and (3) the best approach for incorporating novel mobile health technologies to enhance the goal setting, self-monitoring and peer support elements of the intervention. We aimed to explore the potential role of text messaging, social media, mobile health applications, and photography to enhance the intervention by including demonstrations of sample tools and discussing how to balance in-person group sessions with technology-based activities. Interview protocols were sent to experts for content review and were pilot tested prior to use. Focus group moderators had extensive experience (each with over 10 years) in facilitating groups and qualitative methods.

We held focus groups in collaborating community sites. Discussions were audio recorded and transcribed. The total number of interview hours was 7.26 h. Each adolescent completed a brief questionnaire to obtain basic demographic information. We recorded field notes, reviewed transcripts for accuracy and completeness based on notes taken and imported transcripts into ATLAS.ti (version 8) (ATLAS.ti GmbH, Berlin, Germany) to facilitate coding of text passages and comparison of text within and across groups. Analysis was concurrent with data collection, so that insights from earlier focus groups could inform later groups.

### 2.3. Data Analysis

Two coders (FK and NV) used an open-ended coding approach to independently identify initial codes and then met to iteratively discuss and refine codes. The final codebook contained codes and mutually exclusive code definitions. There was >90% intercoder agreement across the six coded transcripts. Two analysts (NM and NV) next developed memos to interpret and organize focus group content into 15 key topics based on comments made by multiple participants within and across focus groups. The analysts then selected quotations to illustrate and expand upon these key topics and used Thematic Analysis [41] and an iterative process to consolidate findings into three overarching themes with supporting quotations.

## 3. Results

Participant Demographics, Diabetes Related Knowledge, and Preferred Foods and Activities.

Out of the 52 focus group participants (ages 13–22 years), 32 (62%) were male and 20 (38%) were female. Participants self-reported as Latinx/Hispanic (64%) and Non-Hispanic Black (36%).

Teens generally understood that diabetes is a condition that affects blood sugar and insulin, that there are different types of diabetes, that diabetes can be inherited, and that diabetes can be related to being overweight. There were also some misperceptions about diabetes (e.g., one type of diabetes affects blood pressure rather than blood sugar, diabetes affects sodium levels, diabetes can be passed on to someone through physical contact, and diabetes cannot be prevented).

When asked about their favorite food or activity, there was a wide variety of responses. Favorite foods tended to be less healthy while favorite activities included fewer active hobbies (video games, watching shows/movies, music, writing, reading, drawing, singing, taking pictures) and more active options such as sports and dancing.

### 3.1. Overarching Focus Group Themes

Analysis of focus group findings resulted in three overarching themes related to the major domains we explored: influences on adolescent lifestyle, use of a youth peer education model for diabetes prevention, and use of mobile health technologies as part of youth diabetes prevention.

**Theme 1: Adolescents generally encounter unhealthy peer influences related to dietary choices and healthy peer influences related to physical activity.**

### 3.2. Peer Influences on Diet

There were differences in teens’ perceptions of peer influences on their dietary choices. Some teens stated that friends either do not influence what they eat or help them eat healthier at times (Table 1, Quote 1). One participant reported that influence from friends varies based on which friends they are with and can be both negative and positive. However, most teens perceived that their eating habits are unhealthier when they are with friends compared to when they are alone or with family. Factors that cause most teens to gravitate towards unhealthy foods when with friends are the lack of healthy food options in their communities and cost. Teens discussed how there are a wide variety of low-cost fast-food options near their school and that the decision of where to eat is usually influenced by friends (Table 1, Quotes 2–3). Teens stated that friends influence their diet by introducing new foods or sharing meals. For example, some teens said that they feel obliged to eat what their peers are eating even to the point where they feel trapped, are forced to eat certain foods, or develop cravings or addiction to foods (Table 1, Quotes 4–7). In terms of influence of family members, some teens felt that family members are more likely to help control their food intake than friends (Table 1, Quote 8).

### 3.3. Peer Influences on Physical Activity

Teens stated that their friends have a big influence on how physically active they are. A few teens reported that they are less active with friends, and many said that they are often on their phones texting with friends (Table 1, Quotes 9–10). Some teens also mentioned that like dietary behaviors, physical activity behaviors also change depending on which friends they are with (Table 1, Quote 11). However, many teens stated that they choose to spend time with people who are active and involved in sports and that they also encourage their friends to be more active (Table 1, Quotes 12–14). One teen mentioned that peers encourage them to be physically active despite medical conditions such as asthma that may prevent or restrict exercise. Most teens felt that friends generally encourage them to be active and at times even force them to be more active (Table 1, Quotes 15–18). There were mixed feelings about family influences, as many stated that parents encourage them to be active while others ask them to stay home. In comparing influence of family versus friends on physical activity, many teens felt that their family is less active than their friends (Table 1, Quote 19).

**Theme 2: Adolescents endorse a youth peer educator model for diabetes prevention, and describe ideal qualities for peer leaders, strategies for building relationships between program leaders and participants, and methods to motivate, support and evaluate leaders.**

We asked teens about their general perceptions of peer education strategies for youth interventions, desired qualities for peer leaders, how to promote positive interactions between peer leaders and program participants, and how best to support peer leaders.

### 3.4. Peer Education General Perceptions

Teens generally knew what peer education is and liked the idea of peer education for prevention of diabetes (Table 2, Quotes 1–2). Teens gave suggestions for what to include in the program including music related activities, nutrition, healthy snacks, sports/physical activities, trips/outdoor activities, and interactive activities (handouts, discussions, games). When asked about whether peer education programs should be collaborative (participants working together to earn points and prizes) or competitive (competing for points and prizes), most said that a collaborative approach might be better, both because it’s a good way to make new friends and also because some people are too competitive and aggressive. However, for some people competitions are fun and motivate them to work harder. Several teens suggested combining the two strategies (Table 2, Quotes 3–4).

### 3.5. Identification of Peer Leaders and Desired Leader Qualities

Some participants were skeptical about teens listening to other young people and made a point that potential peer leaders would have to be carefully vetted. Teens suggested that peer leaders should have the following qualities: same age as participants or a little older, mature, dedicated, good leadership and communication skills, knowing someone with diabetes, ability to interact with and positively influence others, good personality and character, and can make information fun and interactive (Table 2, Quotes 5–7). Some felt that the leaders should be healthy and fit so that they will be taken seriously in terms of guidance about healthy eating and exercise. Others felt that young people who have struggled with weight or diabetes themselves might be a good role model if they have made changes to be healthier. Teens suggested finding peer leaders in schools, through dissemination of fliers, online, through social media, and through youth-serving organizations. Potential leaders should be interviewed and observed to get a sense of how they would lead the group.

### 3.6. Peer Leader Motivations and Challenges

Teens had suggestions for what would motivate someone to become a peer leader: having friends who are peer leaders, someone they care about having diabetes, improving their own health, gaining experience for a college resume or application, doing something important/making a difference, and having their voice be heard. Teens felt that money should not be a major motivating factor for peer leaders because it is more important that they care about the topic and want to do something positive (Table 2, Quote 8). Teens stated that peer leaders would stick with the program if adolescents attended regularly, were engaged, and participated (Table 2, Quotes 9–10).

Teens were also asked about perceived challenges of being a peer leader. Some common reasons peer leaders might walk away from the role were if they felt like they were not making a difference or that the kids were not learning, if the participants were disrespectful or not listening, if people did not show up to the program regularly, and if the participants and/or leaders were not enjoying it (Table 2, Quotes 11–12).

### 3.7. Peer Leader Relationships with Teens

Teens gave suggestions for how peer leaders can build strong relationships with workshop participants. Some common suggestions included being respectful, building trust, keeping communication open, getting to know the participants on a personal level, showing that they care about the participants and what they are teaching, providing extra help, and being flexible with their schedule (Table 2, Quotes 13–16). Some suggestions for how to stay in touch with the participants included through email and phone calls (less popular), texting, social media, apps (Skype, Google Hangouts, Zoom, Facetime, WhatsApp), as well as being present face-to-face in places where teens usually gather.

### 3.8. Peer Leader Supervision and Support

The teens gave suggestions for how peer leaders should be supervised. The most common suggestion included observation by an adult supervisor (Table 2, Quotes 17–18). The teens also gave suggestions for how peer leaders can be supported during the workshop. Some suggestions included giving them adequate information and materials like checklists and planners, using a buddy system, having a supervisor/teacher conduct regular check-ins and take detailed notes during observed workshop sessions to provide honest and constructive feedback, and providing helpful tips about how to communicate and how to handle challenging situations. (Table 2, Quotes 19–23).

**Theme 3: Adolescents prefer text messaging for motivation and support, and to monitor key diet and physical activity behaviors, track weekly goals, and receive personalized advice and feedback.**

We asked teens about different types of mobile health technologies (text messaging, mobile apps, and social media) and their potential role in a youth diabetes prevention program.

### 3.9. Text Messages

#### Behavior Tracking Messages

Teens were asked if they would be willing to receive text messages to track behaviors such as fruit and vegetable intake, sugary beverage intake, physical activity time, and sedentary activity (screen) time. Teens liked the idea of reflecting on their behaviors, pointed out that these messages could be a useful reminder, and stated that they may share more information via texts than they would out loud in a group setting. Most felt that the best time to get these types of messages would be later in the evening (after dinner and before bed when they are usually on their phones and not very busy, around 08:00 or 09:00 p.m.) (Table 3, Quote 1). Some teens said that they might not pay attention when they are relaxing or may be too tired to text back at the end of the day but acknowledged that they were more likely to do so if they care about being healthy and feel comfortable with the person texting them. While some teens said they would not mind receiving texts more frequently, others recommended that these types of messages could be sent out a few times a week or at the end of the week (Table 3, Quote 2).

Some teens stated that they might not be totally honest if they were engaging in unhealthy behaviors. Others stated that they might not respond if they do not pay attention to everything they eat, or in the case of screen time it may be difficult to answer since people are always on their phones. There was more positive feedback about monitoring physical activity and sugary drink intake (Table 3, Quotes 3–4). Teens stated that it would be important to receive acknowledgment, encouragement, and motivation in an interactive way with these types of messages. Participants suggested that messages be personal and that they give specific recommendations (Table 3, Quotes 5–6). They also liked messages encouraging them to swap out unhealthy foods for healthy foods and to try new foods (Table 3, Quote 7).

### 3.10. Goal-Setting Messages

Many teens said that goal-setting messages were their favorite type of message (Table 3, Quote 8), as they reflected that this would give them something concrete to work towards and that having someone check in with them would hold them accountable and provide support if they are having a difficult time completing their goal. They agreed that messages encouraging goal-setting behaviors would be more effective in motivating them to make lifestyle changes and suggested that visual information about goal completion could help supplement the text messages (Table 3, Quote 9).

Participants identified that messages regarding barriers for goal completion may be helpful for those having a difficult time adhering to their goals (Table 3, Quote 10). However, some teens thought that discussing what is getting in the way of completing a goal might be too complicated to do through texting. As an alternative, a text message could be sent about how the goal is going and then the peer leaders could do individual outreach by phone to discuss in more detail.

Teens stated that the best way to get adolescents to respond to goal-setting messages is to make the messages interactive so that teens feel supported and motivated. They recommended possibly incorporating a competitive component with other participants which could inspire them to do more or rewarding participants with prizes for goal completion. They also came up with creative strategies for getting teens to respond to messages about goals including making jokes or other engaging messages to catch their attention (Table 3, Quote 11).

### 3.11. Motivational Messages

Teens were asked about receiving messages with motivational text and graphics and generally felt that this type of message would be effective (Table 3, Quote 12). Teens could also create their own personalized motivational messages with specific images or words that are meaningful to them (Table 3, Quote 13). They also recommended using inspirational words or quoting someone well known to motivate them (Table 3, Quote 14).

### 3.12. Tailored/Educational Messages

Participants felt that texting educational facts was too simplistic and not very engaging or innovative. Instead, they liked tailored messages (participants are asked a question and get different automated responses back based on how they answer).

Example 1: Ask how often they eat breakfast and if they report that they skip breakfast or eat breakfast infrequently, send back the following: “People who skip breakfast tend to overeat later in the day. Eating breakfast means your energy is steadier through the day and you’ll eat better.”

Example 2: If someone reports that they have felt sad, angry, or frustrated recently, send back the following: “Exercise can make you feel happier. It releases hormones that make you feel good. Try it and see if it works”.

Example 3: For someone who says their cousin is their main support person: “It’s much easier to exercise when someone does it with you. Could you ask your cousin to exercise with you a few days each week?”

They felt these types of messages provided useful information and advice and can serve as reminders of what they should do. Again, they liked personal messages and messages which provided specific suggestions (Table 3, Quotes 15–17).

### 3.13. Timed Prompts/Photo Diary Messages

Teens were also asked about messages sent at certain times of the day or prompting them to send in photos related to healthy eating and active living.

Example 1: “Feeling hungry? What healthy choice can you make for a snack–snap a picture of it and send it in”.

Example 2: “Is this a good time to do something active? What can you do?”

Teens felt it would be good to send these messages after school and around mealtimes to encourage them to make healthy choices (Table 3, Quote 18). Others acknowledged that this could be a good strategy, but that people get out of school at different times and sometimes teens have too much on their mind after school such as homework or spending time with their friends. However, they liked the idea of taking selfies of eating something healthy or doing something active and using photos in general to engage in conversations (Table 3, Quote 19).

### 3.14. Social Media

Teens use social media to keep in touch with people, get information about what is going on locally, catch up on current events, get help with homework, find things to make them laugh, post pictures, and watch videos. When asked about use of social media platforms for a teen health education program, some teens could see the benefit (Table 3, Quote 20). Social media can include things such as daily challenges, healthy lifestyle strategies, polls, community events, information about diabetes, group discussions, photos/videos, interviews, inspirational quotes and games.

Teens were open to the idea of a Facebook page that includes some of the items above, but there was mixed feedback about Facebook. Some criticisms were that you have to go to a separate page for access, that people message and post status updates too much, and that it’s not the best platform to share pictures (Table 3, Quote 21). People mentioned other social media apps, but there was no consensus on using any of them for lifestyle programs.

Most teens said they would use an existing profile rather than creating a new one and also preferred private groups in which only the participants would know of the group’s existence and the members’ identities (Table 3, Quote 22). However, some stated that if we use private groups, we will not be able to involve new people or spread the word about the program. One idea was to allow participants to recommend others to join the group. They would also like it if they could gain more followers. Some teens mentioned that it would be important to have moderators who can keep track and decide what content stays posted on social media platforms (Table 3, Quote 23).

### 3.15. Mobile Health Apps

When asked about apps to track their diet and physical activity behaviors, a few teens mentioned the Nike plus app, Map My Run, Just Dance, and apps to track daily calories. When they were shown examples of other apps used for diet and physical activity tracking, most teens had not tried them before. They liked the idea of apps that allow users to track multiple measures and apps with challenges. Some said they might use these types of apps if they are serious about their health and if they get reminders. However, many said that they are unlikely to keep using these apps consistently (Table 3, Quotes 24–26). Some also reported that they may not remember the foods they ate or tell the truth when recording their food intake. Many felt that use of these apps would likely not inspire them to make changes and recommended focusing on general behaviors instead of detailed tracking and calorie counting (Table 3, Quote 27).

### 3.16. Mobile Health Platform Preferences

When asked about the type of mobile health technology tool they liked the most (text messages, social media or apps), they preferred text messages because accessing messages is simpler and does not require logging onto a separate platform. They also felt that text messages might have more of an impact and be more likely to generate a response than a notification on social media or an app (Table 3, Quotes 28–30). However, participants cautioned that teens might get annoyed if too many messages are sent, and they might ignore messages or even block the number if they are not in the mood to receive messages (Table 3, Quote 31). Most teens were not concerned about security and privacy with mobile health tools, but there were some concerns about potential for cyber bullying. Teens suggested that we should monitor the platform, that participants could go to the leader if someone is getting teased or bullied, or we could block people or make them leave the group if needed.

## 4. Discussion

The objective of this study was to conduct focus groups with youth to explore peer influences on diet and physical activity behaviors which may be associated with diabetes risk, strategies for implementing a youth peer education program for diabetes prevention, and how best to incorporate mobile technologies as part of the program. Our community-based qualitative research approach yielded insights into why youth from urban, ethnically diverse communities are at increased risk for diabetes and how to decrease this risk through a combination of peer education and mobile health technologies.

Some findings from our study have been described previously. Similar to other studies that examined knowledge of type 2 diabetes among at-risk youth [42,43,44], teens in our study had a limited understanding of diabetes, its symptoms and complications, and its connection with diet, obesity, and physical activity.

### 4.1. Social Influences on Teen Diet and Physical Activity Behaviors

Previous studies have clearly documented the impact of peers on adolescent diet and physical activity behaviors [23,45]. In our study, while there were varied perspectives, most teens described negative peer influences on diet and positive influences on physical activity.

### 4.2. Peer Influences on Diet

Past studies have noted that peers influence food choice and shape children’s and adolescents’ food preferences and eating behaviors at a developmental stage when friends gradually become an important source of information about acceptable modes of action [46,47]. Research has shown that children’s and adolescents’ food choices, taste preferences, and actual eating behaviors are influenced by descriptive norms [48,49,50]. In addition, since eating is typically a social occasion, other eaters including peers and siblings, and children’s observations of the eating behaviors of others, influence the development of their own eating behaviors [51].

Some youth in our study stated that their friends can at times encourage them to eat healthier foods. Previous studies have found that adolescents exhibit similarities in healthy eating patterns with their close friends. For example, one study reported peer influences on behaviors such as eating breakfast, eating whole grains, and increased intake of fruits and vegetables [31].

However, most teens in our study preferred less healthy foods, as has also been described in other studies. In general, adolescents view themselves as unhealthy eaters with a normative preference for unhealthy foods [52], and studies show that knowledge regarding healthy food does not counteract this behavior [53]. In addition, most teens in our study reported that their peers tend to negatively influence their diet. Many other studies have observed similar negative influence on peers’ diets [54]. Friends are often seen as barriers for healthy eating and encourage intake of junk foods [55,56,57,58,59]. Our findings align with others who describe how peer considerations may supersede personal judgment to eat well [60], how peer interactions increase both the availability of unhealthy food and the social pressure to eat it [53,56,61,62,63], and how friends may make negative comments when one tries to eat healthy [64]. One novel finding from our study is that many teens described feeling coerced into eating unhealthy foods and becoming addicted to these foods due to influence from their friends; this extreme level of social pressure related to youth dietary behaviors should be explored further in future studies.

### 4.3. Peer Influences on Physical Activity

In contrast to our findings regarding peer influences on diet, we found that peers tend to have a positive influence on youth physical activity levels. These findings are not surprising as research has consistently shown that peer relationships offer important opportunities for companionship and recreation, and that most physical activity during childhood and adolescence, whether organized sports or spontaneous physical activity, involves some form of social play [65]. Along these lines, a number of studies have found that children and adolescents are more physically active when in the presence of peers [65,66,67,68,69], and that youth who report a greater presence of peers in their lives also report engaging in more physical activity [68,69]. One interesting finding from our study, which aligns with some of the findings above regarding peer influences on dietary behaviors, was the perception that peers not only generally influence youth to be more active but sometimes even force them to be active [68].

In contrast, other studies have found that weight criticism and peer victimization may influence children to withdraw from organized physical activity involving peers, such as sports teams, and allocate more time to solitary activities that tend to be sedentary in nature [70,71]. However, peer influences on physical and sedentary activity also seem to depend on which friends teens are spending time with [72,73,74]. For example, teens in our study mentioned that they are more active when they are around friends who are highly active and engage in more sedentary activities when they are with friends who are less active.

### 4.4. Family Influences on Lifestyle Behaviors

Participants in our study also discussed the influence of family members on their diet and physical activity behaviors. Previous studies have identified family support (including cohesion, modeling, and monitoring) as a positive influence on lifestyle behaviors [56,75]. Several studies have recognized that family presence and family meals have a positive impact on teens’ dietary patterns [76,77]. However, youth in our study had mixed feelings about the influence of family members on their behaviors. Most stated that parents encourage them to be active, but some parents asked teens to stay home due to concerns about safety or for help at home. Teens also described how family members sometimes impede healthy dietary behaviors through provision of unhealthy foods, pressure to eat large portions, and lack of support for weight control efforts.

### 4.5. Other Influences on Lifestyle Behaviors

Apart from social influences, teens in our study spoke about other factors that affect their lifestyle behaviors. They commented on disparities in access to healthy food, exposure to advertisements for unhealthy foods, and lack of safe spaces to be active compared with more affluent communities [78,79]. These perceptions certainly have merit, given the abundant evidence that such disparities exist [80,81]. As in other studies, teens in our study identified several additional barriers for healthy eating and active living including taste, time, cost constraints, mood/stress, lack of motivation/energy, and neighborhood factors [52,53,56,60,62,64,82,83,84,85,86,87].

### 4.6. Peer Education for Youth Diabetes Prevention

As it is clear that youth diabetes prevention efforts are needed and that peers have a significant impact on lifestyle behaviors, it follows that peer education programs have potential to improve behaviors and decrease disease risk. Youth peer-led lifestyle interventions have become increasingly common over the last few decades. In general, teens from our study supported the idea of youth peer-led diabetes prevention programs and felt that young people would be more receptive to information provided by peer educators than older adults.

### 4.7. Finding and Training Peer Leaders

Despite overall support for a peer education model for youth diabetes prevention, teens in our study cautioned that potential peer leaders be carefully vetted and demonstrate key qualities (such as being a good communicator, having a personal connection to diabetes, and having certain personality traits such as being fun and dedicated). Along these lines, in a review of youth peer led lifestyle modification interventions, authors highlighted that youth peer educators across many studies underwent various vetting processes, with individual studies using different strategies and criteria to identify and evaluate eligible leaders [88], including age, race/ethnicity, gender, nomination by peers, academic performance, recommendations from teachers and program leaders, and interviews. While teens in our study discussed the need to train peer leaders, they did not provide detailed feedback about training length or content. The review study found that two peer leader training models were associated with improvement in body measures: delivery of the program by teachers to older students who then taught younger students and detailed training of peer leaders prior to program delivery [88]. According to the review, there was significant variation in both the duration of training (ranging from one session to 40 h with ongoing training during program implementation) and the scope of training (including components such as leader roles and responsibilities, review of intervention curricula, team building and group facilitation, and interactive activities) [88]. The review also described recruitment of peer leaders though schools and youth organizations, as suggested by teens in our study. In addition, teens in our study recommended social media as an additional recruitment strategy.

### 4.8. Peer Leader Motivations and Challenges

Teens in our study discussed possible motivations for becoming a peer leader and challenges for staying involved in these types of programs. The most important motivating factors included a personal interest in diabetes/health, wanting to help others, and gaining leadership experience including for college applications and resumes. The most cited challenge for continued peer leader involvement in programs was perceived lack of engagement from program participants. We have not found other studies that explored potential motivating factors and barriers for young people who have considered becoming a peer leader or who have served as peer leaders. Thus, the findings from our study fill an important gap in the literature and may assist others interested in employing youth peer leaders in lifestyle modification programs.

In addition, even though prior studies did not explicitly assess peer leader motivations, some studies have highlighted positive impacts of youth peer led lifestyle interventions on peer leader health behaviors and outcomes. For example, one study focused on improving diet behaviors in school cafeterias found that students trained as peer advocates had improvements in sugar sweetened beverage intake [89]. Another found that participation as a peer communicator in a school-based intervention led to a lower percentage of obesity compared to a control group [90]. Similarly, another study found that students who were highly involved in a peer-led health promotion intervention were more likely to report healthy eating behaviors and positive attitudes to low-fat foods [91]. Thus, peer leaders might be motivated to stay involved with programs if they note improvements in their own health as they are trying to help others.

### 4.9. Peer Leader Supervision and Support

Teens in our study suggested that peer leaders should have an adult supervisor who can give them feedback, ongoing suggestions for improvement, and checklists or other materials to support them. The previously published review of youth peer-led lifestyle interventions described how many interventions included supervision of youth peer leaders by adult research staff or site leaders [85]. Feedback and support was generally provided to peer leaders on an individual basis with varying frequency, but some studies also included group check-ins with peer leaders during the intervention. In addition, most interventions that reported improvements in weight related outcomes included supervision of leaders on at least a weekly basis.

### 4.10. Use of Mobile Technologies for Youth Diabetes Prevention

Another aspect to consider in developing youth prevention programs is how best to keep participants motivated and engaged. The use of mobile and wireless technologies and wearable devices for improving health care processes and outcomes (mHealth) has generally been shown to be promising for health promotion among patients with chronic diseases such as obesity and diabetes [92]. We thus explored the potential role of different types of mHealth tools to support and engage teens as part of broader youth diabetes prevention efforts.

### 4.11. Social Media

While some teens in our study could see the potential benefits of using social media platforms for youth health education programs, there were mixed feelings about Facebook and no consensus on use of other platforms. Some of the major reasons cited for not employing social media as the primary technology component of such interventions were the need to log in to a separate website or app to access content, concerns about the appropriateness of content or too many messages being posted by participants, and difficulty keeping teens engaged over time.

Other studies have also noted challenges with social media-based lifestyle programs. A recent systematic review of the use of social media in nutrition interventions for adolescents and young adults found that several studies reported declines in interactions over time and low participation in features such as blogs, discussion boards, and recommended peer chats [93]. For example, in a sample of college students, engagement in a social media weight control intervention was variable, and engagement tended to decline over time [94]. In another study, although there were high rates of initial interest (78%) in a Facebook weight control intervention, only 14% of those indicating interest (8% of total sample) actually joined the group [95]. The systematic review also concluded that adding social components to behavioral interventions does not always lead to high user engagement because individuals are more likely to use social media sites to maintain existing social networks, rather than to develop new relationships [93].

### 4.12. Mobile Health Apps

Most youth in our study had not used mobile health apps to track their diet and physical activity behaviors. Many said that they might try these types of apps, especially if they included challenges, but that it was unlikely that they would accurately and consistently track their behaviors. Teens also felt that use of apps requiring detailed behavior tracking would likely not inspire them to make changes and instead recommended focusing on general healthy behaviors.

Other studies have examined the impact of mobile health apps on lifestyle and health related outcomes in youth. A systematic review found that the use of a mobile app, with or without another intervention, did not result in any significant differences in anthropometric measures (BMI, waist circumference or percent body fat) [96]. While improvements in knowledge, attitudes, and motivation were reported in some studies, results regarding changes in self-efficacy to engage in healthy behaviors were mixed. There does seem to be some promise for use of apps to improve goal setting and nutritional behaviors, but changes in physical activity levels and screen time associated with interventions using a mobile app varied [96]. The review concluded that personal characteristics may limit the effectiveness of potential apps, and that app-related competition may not always be a positive force for behavior change [96].

### 4.13. Text Messaging

#### 4.13.1. General Feedback

When asked which type of mobile health technology tool they preferred for use as part of a youth diabetes prevention program, most participants stated that they liked text messaging the most because information is more easily accessible. They also felt that text messages might have more personal impact and be more likely to generate a response than a notification on social media or an app. Along these lines, previous literature has shown text message interventions to be acceptable and effective in improvement of lifestyle behaviors in youth [97,98,99]. In addition, in contrast to social media and mobile app-based interventions, text message interventions have been shown to positively impact clinical outcomes. In a recent systematic review of the effectiveness of text message interventions for weight management in adolescents, seven out of eight studies demonstrated reductions in BMI or BMI z-score in the intervention group compared with the control at the end of the final follow-up [100].

#### 4.13.2. Message Types

Teens in our study especially liked text messages that allowed them to reflect on their behaviors and track them over time, messages that helped them set and track goals, and messages which provided specific, personalized suggestions. Previous studies have found that the use of gold standard behavioral approaches has been associated with improved outcomes, including using such evidence-based weight control strategies as goal setting and self-monitoring [101]. Some form of personal contact also appears to enhance outcomes, including support and personalized feedback on progress [102]. Individually tailored and personalized motivational text messages have also been shown to be feasible and acceptable and may positively impact behaviors [102,103].

#### 4.13.3. Gaps in the Literature/Next Steps

Studies to date focused on text messaging to improve lifestyle behaviors among youth have included text messaging combined with online education modules, wearable devices/behavior tracking apps, and in-person individual or group sessions with health professionals [100]. None have used peer education or videoconference platforms for program delivery.

The recent systematic review of the effectiveness of text message interventions for weight management in adolescents found that only half the studies used two-way text messages (participants able to respond) and personalized text messages [100]. Only one study detailed that text messages were sent from an automated computer system capable of tailoring the text messages using an algorithm based on participant feedback via text message [104]. In this study, intervention participants demonstrated significantly higher self-monitoring adherence, but participants reported that they would prefer fewer and more personalized text messages [104]. The review concluded that future interventions should consider interactive two-way text messages as an intervention delivery strategy for adolescent populations [100].

In addition, few studies have engaged adolescents in text message development. The most comprehensive text message development with adolescents involved testing text messages in a 3-month feasibility study, surveying adolescents about text message content, timings, and interactivity at monthly intervals, and conducting semi structured interviews at the end of the study [104]. This participatory development process allowed the researchers to develop a text message bank that was engaging for participants, with 85% of participants finding the text messages helpful. Similarly, another feasibility study utilized extensive participatory methods with adolescents to develop 300 healthy lifestyle messages and a delivery protocol [105]. The previously published review concluded that co-design may increase the likelihood of acceptable text messages and result in effective engagement in future interventions for adolescents [100].

### 4.14. Limitations

There were several study limitations. Individuals may not fully disclose information in group settings and participants may be unduly influenced by the ideas of others. Although theoretical saturation was achieved, the moderate sample size may not have allowed recognition of all relevant ideas. Developmental and other differences based on participant age may have affected results. However, the authors decided to target adolescents with the plan that youth from similar backgrounds would later be the focus for the developed program. Youth leaders in partner organizations helped recruit participants, which may have skewed the participant sample, affecting generalizability. For example, these results may not be extrapolated to adolescents from other countries and backgrounds. However, this study was designed to inform a youth diabetes prevention model for this specific community, rather than to define beliefs in the general adolescent population.

### 4.15. Adaptation of Youth Diabetes Prevention Model

Using study findings, our CAB developed a diabetes prevention program for prediabetic adolescents. Focus group findings were translated into program components (Table 4).

## 5. Conclusions

In summary, our qualitative research approach yielded important insights into why youth from urban, ethnically diverse communities may be at increased risk for diabetes and how to decrease this risk through a combination of peer education and mobile technologies. We used findings to create a program that can be sustained by the community and adapted and disseminated to other high-risk communities.

## Figures and Tables

**Table 1:** Representative Subthemes and Supporting Quotes for Theme 1: Adolescents generally encounter unhealthy peer influences related to dietary choices and healthy peer influences related to physical activity.

Quote Number	Quote
**Peer Influences on Diet are Mixed but Largely Unhealthy**	
1	“Since me and my friends, we play sports, they notice how slow I get and they try to encourage me and tell me you’re running slow, you have to eat healthier.”
2	“How my friends influence what I eat is like-at lunch me and my friends start getting the same exact thing that we order. We all get the same thing together…o we usually eat like pizza, fries or like…and get two of those and a soda.”
3	“No, after school with your friends if that’s like the scenario, if you’re with your friends and they’re eating at McDonalds you’re going to be like no I’m going to pass, I’m going to get a salad. Are you going to do that around your friends?”
4	“Like if you’re with a group of people and they all decide to go get one specific thing and you want something else it might cause you like to change your mind and just get what they’re getting.”
5	“My friends make fun of me…like if I get something…they’re like what are you getting, you can’t get that, get something else. So I get it.”
6	“…if I say I’m craving something then she wants it too. If she says it then I say it too.”
7	“Oh, my friends, they’ll be like oh you have to try this, this is really good, and then they get me hooked and I can’t live without it. It’s horrible.”
8	“So when I’m with my friends and stuff I eat a lot of junk food like chips and bread but when I’m with my family I eat a lot of white rice and they help me cut down.”
**Peer Influences on Physical Activity are Mixed but Largely Healthy**	
9	“All my friends are lazy so we don’t really do much. When I’m with them we don’t do much activity at all, yeah.”
10	“Yes because they’re lazy and like I [would like to] go do something…and they’re like well we’re going to go inside.”
11	MODERATOR: So for some of you sometimes depending on who the friends are? MALE VOICE: Yeah like some of my friends are gameheads so if I’m hanging out with them- MALE VOICE: (interposing) You mean geeks? MODERATOR: Then you’ll be more likely to sit around? MALE VOICE: Yeah, but if I play with my football friends then… MODERATOR: More likely to be active.
12	“Like when I go to school I’ll go to the weight room with my friends and my friends are like very competitive, athletic kids so like they make me want to be better than them and…competition.”
13	“After school if I’m bored or something I’ll go with my friends to the park and we’ll play basketball or we’ll just run.”
14	“My friends are too active. They’re like junkies. They’re like junkies…buff muscles. That’s my friends. They’re junkies.”
15	“My friends keep me from being lazy. Every day we walk to school instead of taking the bus and whatnot.”
16	“I think my friends definitely…influence me but they wouldn’t want me to go-for example last year, actually this year I joined track because I had a friend on the team and they were like yeah, just come and…and you’ll like it so I did.”
17	“Alright so today I wanted to be lazy at lunch and I was just going to sit on the bench and they’re like come on BOY 1, we need another person…play a game. So I got up and I played basketball.”
18	“And if they choose to play then you got to play.”
19	“My parents are lazy. They don’t do anything. So I usually just work out or like on the weekends just play football. That’s when I’m with my friends.”

**Table 2:** Representative Subthemes and Supporting Quotes for Theme 2: Adolescents endorse a youth peer educator model for diabetes prevention, and describe ideal qualities for peer leaders, strategies for building relationships between program leaders and participants, and methods to motivate, support and evaluate leaders.

Quote Number	Quote
**Peer Education General Perceptions**	
1	“It’s more like when people our age talk to us we listen more and with adults it’s basically like okay we listen to you but we don’t really want to understand or don’t really want to do what you’re saying. So like when it’s somebody your age you kind of listen and you kind of do what they’re saying and take it into consideration.”
2	“I think it’s a good idea because people of our age have more of an influence than older people so we’d be able to influence people our age to do more stuff.”
3	“I like teams working together because if you do lose or you don’t get whatever you wanted at least you will have an experience working with somebody because if you just compete at the end of the day it looks like you’re just there for one thing and one thing only but if you work together at least you made new friends.”
4	“Maybe in both kind of because if you’re competitive that makes you work harder but at the same time if you don’t win then maybe you’ll get angry. If you work together then you’re not really working on the negatives of how somebody reacts to losing, you’re just working on doing points and everything.”
**Identification of Peer Leaders and Desired Leader Qualities**	
5	“Kind of like a role model or an experienced person with diabetes or a person that already has learned about it or has seen somebody go through it.”
6	“Someone just like naturally a great leader and could kind of influence you, so like a good speaker.”
7	“I think we should just find someone who is very informational about it and could also give a very good presentation and also make it very interactive and also fun.”
8	“Being able to help somebody…I know people who had really difficult challenges and I’ve experienced a little…I wouldn’t want anybody else to live like that so being able to just spread that information of how to prevent that.”
**Peer Leader Motivations and Challenges**	
9	“If I see kids-the information that I’m giving them…apply it to their lives, like if I see them drinking water more or exercising more or eating more healthy then I’ll continue doing it.”
10	“I would stick with it if like everyone was enjoying it or I felt important like I was actually doing something.”
11	“If I’m a peer leader and everyone seems to not care about the topic, like I understand when you first get here you’re not going to care about it if you don’t know about it, but after I have taught my lesson and whatever you still don’t care about it you won’t see me again.”
12	“If I didn’t feel like anyone was enjoying it and I wasn’t engaging anyone I wouldn’t really try anymore.”
**Peer Leader Relationships with Teens**	
13	“It’s not that hard to build relationships with teens. I guess people think too much about it. They’re nice and cool and you do fun things, that makes me want to like you and you don’t have an attitude or anything like that. It’s pretty easy.”
14	“Showing that they care about what the person is going through or about what they’re talking about, showing that they actually care about what they’re teaching.”
15	“I would try to speak to them how I would want to be spoken to and in a way they understand because I’m not going to sit there like I’m smarter than them or anything trying to teach them things that they probably don’t already know. Like I’ll sit there with them, be like-like I’ll say it to them in a way they understand, like I will say like-like throw a little slang in there or like something so they understand like what I’m trying to say to them.”
16	“Actually show an interest in what the kid has to say…because you can always tell when the kid has a real strong connection with an older classmate or something.”
**Peer Leader Supervision and Support**	
17	“Maybe another adult that’s not really-that doesn’t really go in and disrupt what they’re doing but watches what he’s teaching, he or she is teaching and how he does it or she does it.”
18	“You should just have-you see how she’s sitting in on our conversation, have someone sit in on the conversation to make sure they’re doing their job and if they ever go off-topic you either wait until the end of class or pull them aside.”
19	“…the person who is like adult. Just always be there just in case.”
20	“Yeah, I think that you should guide them throughout the whole program so they can know which-where they’re doing a good job or not so that they can change something if something is going wrong. MODERATOR: So giving them feedback. MALE VOICE: Yes, feedback.”
21	“You could take a survey of students, like ask them-like take a survey on a handful of students and ask them how class went.”
22	“To make sure, check and see if the people are actually learning something in the class.”
23	“Well they could have like one supervisor that will probably come in and take notes but the leader should be trustworthy where they don’t actually need someone to…someone high up to supervise.”

**Table 3:** Representative Subthemes and Supporting Quotes for Theme 3: Adolescents prefer text messaging for motivation and support, and to monitor key diet and physical activity behaviors, track weekly goals, and receive personalized advice and feedback.

Quote Number	Quote
**Text Messaging—Behavior Tracking**	
1	“Yeah, I feel like there are some people that are probably on their phones at this hour and they’re going through that I can’t see phase so they’re like let me reflect on what I did today because I have nothing else to do because I’m bored and I can’t sleep. So they’re going to think about all the things that you just sent them because that’s the most relevant thing that’s come to them at a certain time, especially before bed. Because I don’t know if you’re like me but most times before I go to sleep I think about everything I’ve done for that day and it’s like if somebody sends you something like this I’m going to be like okay, maybe I should think about what I’ve eaten…ecause I didn’t eat at all today.”
2	“I don’t think every day because I feel like every day, then you will get tired…you start ignoring them after a while.”
3	“I think what type of physical activity you did, that’s a good question. So like did you go to the gym or I don’t know, play basketball or do some type of physical activity.”
4	“And how many drinks, sugary drinks you did, that’s a really good one because you had like a can of soda or I don’t know, a juice, something like that.”
5	“It’s actually pretty good because it’s actually telling you what you could do and what you-what some examples of what you can do. Like some people just don’t eat fruit but the text is actually letting you know that you can do it in different ways.”
6	“They should also have steps on how to make a smoothie or like yogurt because if-me personally if a friend went to make a smoothie or a yogurt and I don’t know how to do it I would like… FEMALE VOICE: I’d skip it and would go get something else MODERATOR: You wouldn’t try it if you didn’t know how to do it? MALE VOICE: Yeah. MODERATOR: So giving directions almost like a recipe of how to-and directions how to make it would be helpful? FEMALE VOICE: And for the exercises what kind of exercises you want we can do together”
7	“I like the trying something new because it’s kind of telling you you should just not stick to like everyday stuff, not just do this one thing over and over again, try something new.”
**Text Messaging—Goal Setting**	
8	“I like those week goals or those monthly goals instead of like day-to-day goals.”
9	“…you gave me a goal reminder and it was like…walk 10 blocks three times a week I would be like oh yeah I have to remember that and I can do it and I get like a visual on my progress.”
10	“I think that’s a good idea. Like the part where it says what is getting in your way of completing your goal so you can figure out what you need to change so that you can complete your goal.”
11	“You got to trick me into it. If you trick me into it I’m going to laugh at it. You got to say something funny to set it up, you got to be like did you do it and then…be like did I do what? Did you do your goal and I’m like oh they got me and I’m going to start laughing…and I’m going to do extra.”
**Text Messaging—Motivational Messages**	
12	“Maybe a picture of your favorite food or something that’s healthy like an apple or a pineapple.”
13	“Like for me you would send me is football really life and you would send a picture of my favorite team the Ravens and it would motivate me because I feel like I would look at them and be like oh snap that’s my team and then I’m going to have to play football at that moment.”
14	“Because when you’re thinking of something you heard that’s making you think maybe I should have tried it; maybe I should have done it.”
**Text Messaging—Tailored/Educational Messages**	
15	“…they remind you about stuff because you probably just don’t even think about not eating or the way you eat until you get a text message about it and then you’ll think about it.”
16	“I like this way. It’s more personal. It’s giving you specific things that you could do.”
17	“Because like say if you said to get more active but I didn’t know what to do to get active, you gave me suggestions maybe I could ask my cousin to work with me. It gives ideas so I know what to do.”
Text Messaging—Timed Prompts/Photo Diary Messages	
18	“I think after school is like the perfect time because like what she said, people go to the store and make unhealthy decisions. That happens to me every day. So like that’s the perfect time to text a kid make healthy decisions.”
19	“…be engaged in conversation by sending you guys a picture of what we ate. That would be cool.”
**Social Media**	
20	“Yeah, I think it’s a good idea because you get to compare what you’re doing with other people and maybe find other strategies to keep a healthy diet or stuff like that, just share…so that more ideas can come up…more solutions to a certain problem can be come up with.”
21	“Facebook is just too much…People they argue, post they’ve got problems, everything. Like I’m going to the hospital today to get my flu shot. Nobody needs to know all that. Keep that to yourself.”
22	“Yeah, and post it on a private page…private page. It stays relevant to the people inside of the group rather than posting it on your personal Facebook page.”
23	“As long as there’s someone keeping an eye on it and regulating it and it’s a safe environment for people to post things on the page and stuff like that.”
**Mobile Apps**	
24	“No, I’ll probably do it once in a while. Like I would do it one day or one week and then I’ll like skip a whole week and then I’ll do it the next week.”
25	“Because it’s too much work? FEMALE VOICE: Yeah…Like I could be wanting to do something and then I’ll get distracted, oh let me see what’s on Instagram, let me see…and I just forget all about it.”
26	“You don’t think people would get sick of entering their information day after day after day? Yes they would…after a week.”
27	“I don’t like apps that control how you eat. I don’t think that they’re good for you because if you look at the food through calories I think it’s not a smart way to do that. I think it’s better to know what you’re eating and just know that…cut certain things, like cut soda, cut junk food…instead of counting calories because counting calories restricts you from other things that may not necessarily be unhealthy because…because of the…calories…from the point of view of whether it’s-yeah, I think just counting calories is bad.”
**Mobile Health Platform Preferences**	
28	“For some reason, I don’t know, for communication purposes I think text messages impact me the most. If I get a text I think I’m more likely to respond to it than a message on Facebook.”
29	“Because texting is easier than social media and because I like texting a lot and I do it. Sometimes I’m on social media, sometimes I’m not so I’m not always going to respond to that.”
30	“-it’s sending you direct texts on like what you should do and what things you should think about and stuff like that.”
31	“It might be ineffective because if you keep sending texts like every day people might get annoyed.”
32	“Some people might actually care about cyber-bullying because some people might take what people say into consideration…Like it might get to them and hurt them and make them feel bad.” MODERATOR: “So you think it’s important for it to be monitored? FEMALE VOICE: Yeah.”

**Table 4:** Translation of Focus Group Findings into Components of Youth Diabetes Prevention Program.

Focus Group Finding	Strategy for Incorporation into Program
**Theme 1: Peer Influences on Diet and Physical Activity Behaviors**	
Teens generally encounter unhealthy peer influences related to dietary choices (and are sometimes “forced” into unhealthy dietary behaviors)	Role play—communication skills with friends and family including the “broken record” method, “I” messages, expression of feelings, listening, and repetition
Teens generally encounter healthy peer influences related to physical activity	Group based physical activity with music and modeling by peer leaders; friendly competition for step counts
**Theme 2: Peer Education for Youth Diabetes Prevention**	
Workshop Structure	12-week group peer led program (1.5 h per session, held once a week)
Interactive Workshop Activities	- Group exercise demos with music - Games (focus on topics related to healthy eating and being physically active; modeled after popular games; new teams formed each week; collaborate to earn points; top 3 receive a prize at the end of the workshop) - Weekly goals - Group discussions (focus on topics related to healthy eating and being physically active *; brainstorming, problem solving, role plays)
Identification of Peer Leaders	- Distribution of applications through community partners - Individual and group interviews - Selected individuals with desired qualities described in focus groups
Peer Leader Training	- Review of 12 weeks of workshop content, team building activities, and leadership/group facilitation skills (how to handle challenging situations, being flexible, dealing with conflicts, providing feedback, modeling, how to deal with downtime, and preparing/planning)
Peer Leader Supervision	- Observation of sessions by adults - Observation checklist and feedback forms completed for each session
Peer Leader Support	- Weekly planning and debriefing meetings before and after each session - Individual and group feedback
**Theme 3: Mobile Health Technologies—two-way, personalized, interactive text messages**	
Youth Engagement in Platform Development	Use of a participatory process with youth to codesign platform structure and message content.
Text Messaging—Behavior Tracking	- Messages about key diet and physical activity behaviors (fruit and vegetable intake, sugary beverage intake, physical activity time, and screen time) with automated messages sent back to participants based on their response - Workshop specific behavior tracking message based on topic covered during workshop that week with automated messages sent back to participants based on their response
Text Messaging—Goal Setting	- Goal-setting message sent at the beginning of the week - Mid-week goal check in message with automated responses sent to participants based on whether they report their goal is going well or not. Flag participants struggling with their goal for individual outreach from peer leader - Goal completion message at the end of the week with automated responses sent to participants based on whether they report that they completed their goal. Flag participants who do not complete goal for individual outreach from peer leader
Text Messaging—Motivational	- Link with motivational graphic and text OR inspirational quote
Text Messaging—Tailored Messages	- Messages sent to participants asking about specific behaviors - Automated messages sent back to participants based on their response, including specific suggestions with links
Text Messaging—Photo Diary	- Teens instructed to take and upload photos related to healthy eating or active living
Message Frequency	1–2 messages per day
Monitoring of Platform	Centralized message tracker which documents messages sent and received and allows research staff/peer leaders to monitor messages, manually initiate messages, and respond to flagged messages

* also include topics such as stress and dealing with difficult emotions, body image, healthy and unhealthy weight control behaviors, mindfulness, positive thinking, youth empowerment/advocacy, local resources, and healthy lifestyle on a budget.
